# Compound Heterozygous *PIGS* Variants Associated With Infantile Spasm, Global Developmental Delay, Hearing Loss, Visual Impairment, and Hypotonia

**DOI:** 10.3389/fgene.2020.00564

**Published:** 2020-06-16

**Authors:** Lily Zhang, Xiao Mao, Hongyu Long, Bo Xiao, Zhaohui Luo, Wenbiao Xiao, Xingbing Jin

**Affiliations:** ^1^Neurology Department, Xiangya Hospital, Central South University, Changsha, China; ^2^Department of Medical Genetics, Maternal and Child Health Hospital of Hunan Province, Changsha, China; ^3^Neurosurgery Department, Xiangya Hospital, Central South University, Changsha, China

**Keywords:** glycosylphosphatidylinositol, congenital disorders of glycosylation, phosphatidylinositol glycan class S, infantile spasms, whole-exome sequencing, intellectual disability

## Abstract

Glycosylphosphatidylinositol (GPI) is a membrane anchor for cell surface proteins. Inherited GPI deficiencies are a new subclass of congenital disorders of glycosylation. Phosphatidylinositol glycan class S (PIGS) is a subunit of the GPI transamidase which plays important roles in many biological processes. In this study, we present a Chinese boy with infantile spasms (ISs), severe global developmental delay, hearing loss, visual impairment (cortical blindness), hypotonia, and intellectual disability and whose whole-exome sequencing (WES) identified compound heterozygous variants in *PIGS* (MIM:610271):c.148C > T (p.Gln50^∗^) and c.1141_1164dupGACATGGTGCGAGTGATGGAGGTG (p.Asp381_Val388dup). Flow cytometry analyses demonstrated that the boy with *PIGS* variants had a decreased expression of GPI-APs. This study stresses the importance of including the screening of *PIGS* gene in the case of pediatric neurological syndromes and reviews the clinical features of *PIGS*-associated disorders.

## Introduction

There are more than 150 different eukaryotic cell surface proteins attached to the plasma membrane by glycosylphosphatidylinositol (GPI). GPI anchoring is critical for the expression of those proteins, including adhesion molecules, receptors, and enzymes, on the cell surface (Fujita and Kinoshita, 2012). The GPI transamidase replaces a protein’s C-terminal GPI attachment signal peptide with a preassembled GPI, which mediates GPI anchoring in the endoplasmic reticulum (Ohishi et al., 2001, 2003). Phosphatidylinositol glycan class S (*PIGS*) encodes an essential component of the GPI transamidase that mediates GPI anchoring in the endoplasmic reticulum (Ohishi et al., 2001). Neurological syndrome, including seizures, intellectual disability, muscular hypotonia, and multiple congenital malformations, was reported in the majority of individuals who had defects in the GPI–anchor–biosynthesis pathway.

Infantile spasm (IS) is an epileptic syndrome occurring in children younger than 1 year, which can be due to genetic, metabolic, or structural conditions (Hrachovy and Frost, 2013). Increasing reports have shown that IS could be caused by variants of genes involved in the pathways for brain function and development (Hancock et al., 2013). IS is characterized by epileptic spasms, developmental delay, and a specific brain wave pattern on electroencephalography (EEG) called hypsarrhythmia (Hrachovy and Frost, 1989).

Until now, only one article reported six patients with *PIGS* variants (Nguyen et al., 2018). Here we describe novel pathogenic variants of *PIGS* in a Chinese boy with ISs, severe global developmental delay, hearing loss, visual impairment, hypotonia, and intellectual disability, which summarized the clinical features of *PIGS*-associated neurological disorders.

## Case Report

**FIGURE 1:**
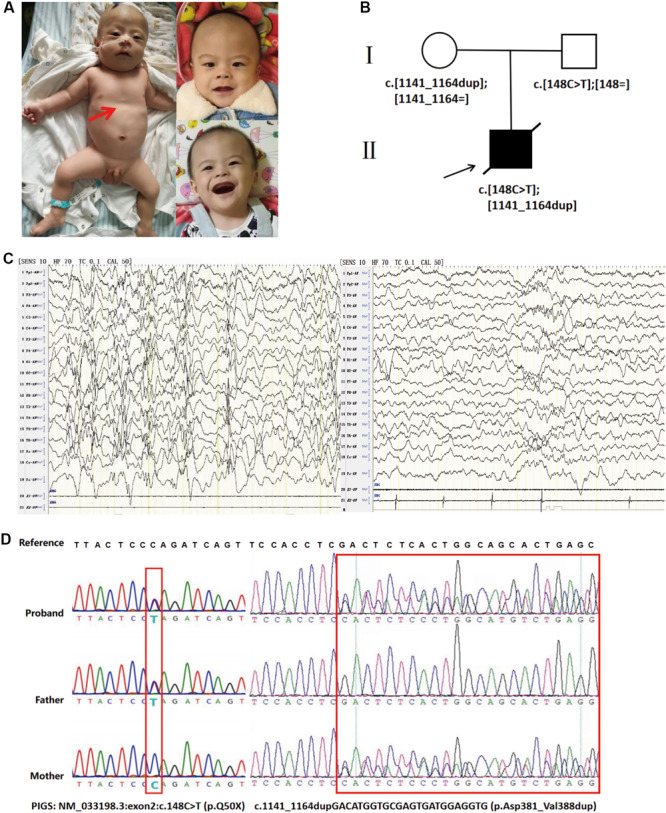
**(A)** Photographs of the affected individual. Funnel chest of the affected individual (left). Coarse facial features of the affected individual at the age of 13 months, including almond-shaped palpebral fissures, arched eyebrows, long nose, deep philtrum, thick helices, no teeth, and gingival hypertrophy (right). **(B)** Family pedigrees: *PIGS* variants in this family as demonstrated by whole-exome sequencing. **(C)** Interictal electroencephalogram (EEG) at the age of 8 months showed hypsarrhythmia consisting of bilateral, high-amplitude, irregular slow waves mixed with multiple focal spikes or polyspikes in the interictal period (left). The EEG of an age-matched healthy control. **(D)** Sanger sequencing electropherograms showing both variants in *PIGS* (right).

The patient was the first child born to non-consanguineous Chinese parents and was a full-term newborn by vaginal delivery. There was no perinatal brain injury, hypoxia, or infection of the central nervous system. There was no family history of epilepsy or other neurological conditions. He had IS when he was 2 months, the flexor spasm of his upper limbs occurring in clusters, and head turning, and his interictal EEG revealed hypsarrhythmia. Seizure frequency was up to 10 seizures per day and was aggravated by fevers or infections. Multiple anti-epileptic drugs, including sodium valproate (60 mg/kg/day) and levetiracetam (50 mg/kg/day), have been given to the boy at the age of 3 months, but the seizures were not well controlled, occurred about once a week, and were partly evolving into status epilepticus. At the age of 11 months, low-dose adrenocorticotropic hormone therapy (20 IU/day) was started, combined with valproic acid administration, but the seizures still occurred two or three times every month. On the last examination at age 13 months, he has severe hypotonia with decreased deep tendon reflexes and profound developmental delays. He was unable to smile, track, hold up his head, and sit independently at 13 months. In addition, he was noted to have coarse facial features, including almond-shaped palpebral fissures, arched eyebrows, long nose, deep philtrum, thick helices, no teeth, and gingival hypertrophy. He also had funnel chest and short metatarsals, but his height and weight were within the normal range (Figure 1A). However, he had feeding difficulties and began to have frequent episodes of vomiting. The recurrent vomiting prompted recurrent respiratory infections. The child died at the age of 15 months due to asphyxia during an epileptic seizure. Autopsy was not performed.

Prior laboratory tests of this patient included normal karyotype, a normal newborn screen, normal urine organic acids, and negative urine mucopolysaccharidosis screen. Brain computed tomography and magnetic resonance imaging (MRI) were unremarkable at 5 months. However, brain MRI of 1.5 T at the age of 13 months indicated mild cerebellar atrophy. Visual evoked potential indicated cortical blindness. Besides that, brainstem auditory evoked potential demonstrated that he had bilateral hearing loss. Interictal EEG at the age of 8 months showed hypsarrhythmia consisting of bilateral high-amplitude irregular slow waves mixed with multiple focal spikes or polyspikes in the interictal period (Figure 1C).

## Results

### Gene Sequencing

We performed whole-exome sequencing (WES) of peripheral-blood DNA samples from the patient and his unaffected parents. The proband’s WES identified compound heterozygous variants in *PIGS* (NM_0033198.3: c.148C > T at exon2 and c.1141_1164dupGACATGGTGCGAGTGATGGAGGTG at exon10). The truncation variant c.148C > T (p. Gln50^∗^) was inherited from the proband’s father, while the in-frame duplication was inherited from the mother (Figure 1B). Sanger sequencing was used to confirm the identified *PIGS* variants (Figure 1D). In addition, the *PIGS* variant c.1141_1164dupGACATGGTGCGAGTGATGGAGGTG was predicted to be deleterious in Provean, with a score of -12.124. Based on the clinical manifestations of the proband and the principle of familial co-segregation, other variants of the proband identified by WES were considered as non-pathogenic.

### Flow Cytometry

**FIGURE 2:**
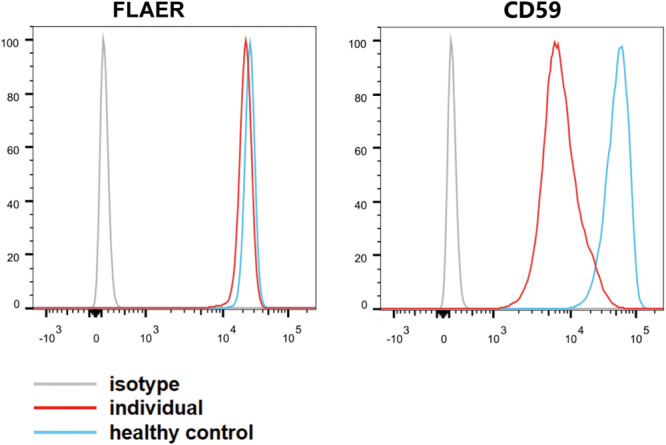
Fluorescence-activated cell sorting analysis of glycosylphosphatidylinositol-APs on individual granulocyte cell surface. The blood granulocytes from the affected individual (red line) and a healthy control (blue line) were stained with GPI-AP markers (CD59–FITC) and fluorescence-labeled aerolysin. The light gray line represents isotype.

Flow cytometry was performed with peripheral blood cells collected from the affected individual and an age-matched control. Blood cells were stained the mouse anti-CD59 (FITC) and Alexa 488-fluorochrome-conjugated aerolysin (FLAER; Cedarlane, Canada), which specifically bind to GPI anchors. The cells were analyzed by flow cytometry (Canton II; BD Sciences, NY, United States) and FlowJo software (FlowJo, LLC, OR, United States). Fluorescence-activated cell sorting analysis on granulocytes demonstrated that the individual cells had less signal of CD59 than the healthy control cells, and the level of FLAER in granulocytes was slightly downregulated (Figure 2). Thus, our study indicated that the pathogenic variants c.148C > T (p.Gln50^∗^) and c.1141_1164dupGACATGGTGCGAGTGATGGAGGTG (p.Asp381_Val388dup) in *PIGS* lead to low amounts of GPI-APs in peripheral white blood cells.

## Discussion

With the rapid development of next-generation sequencing and the widespread application of WES and whole-genome sequencing, increasing reports indicate that variants affecting proteins related to the synthesis of the GPI anchor cause a series of overlapping phenotypes, including seizures, dysmorphic features, muscular hypotonia, and severe intellectual disability (Nguyen et al., 2018). These disorders result from the failure of the GPI transamidase complex, including PIGS, to transfer the GPI anchor to the precursor protein bearing a GPI attachment signal sequence.

There are at least 26 genes involved in the biosynthesis and the remodeling of GPI-anchored proteins. Among these genes, *PIGY*, *PIGW*, *PIGG*, *PIGM*, *PIGV*, *PIGN*, *PIGL*, *PIGA*, *PIGO*, *PIGT*, *PIGC*, *PIGQ*, *PIGP*, *PGAP1*, *PGAP2*, and *PGAP3* variants were reported to be related to human genetic diseases (Freeze et al., 2014). A hypomorphic promoter variant in *PIGM* leads to inherited GPI deficiency, characterized by a propensity to venous thrombosis and absence seizures (Almeida et al., 2006). Germline variants in *PIGA* with developmental arrest, ISs, contractures, dysmorphism, elevated alkaline phosphatase, and mixed hearing loss were reported by Tarailo-Graovac et al. (2015). Early-onset ISs appear to be a common manifestation of individuals with defects in GPI anchor biosynthesis. Additionally, hypsarrhythmia EEG was reported in IGDs caused by disease-causing variants in *PIGA*, *PIGP*, and *PIGW* (Chiyonobu et al., 2014; Tarailo-Graovac et al., 2015; Vetro et al., 2020). Yang et al. (2018) reported a homozygous *PIGT* variant in a Chinese boy with multiple malformations, hypotonia, seizure, and profound developmental delay. Throat–trachea–bronchial softening was observed in the patient at 5 months of age, leading to expectoration difficulty and recurrent respiratory infections. PIGS and PIGT are members of the GPI transamidase complex and have been demonstrated to be essential for the formation of carbonyl intermediates during the transfer of the GPI group to the protein (Ohishi et al., 2001). It is worth noting that airway softening may be the potential cause of recurrent vomiting and respiratory infections in our patient. To our knowledge, this is the second clinical report associated with *PIGS* variants. Nguyen et al. (2018) have reported six patients, from three unrelated families, with *PIGS* variants in 2018. Furthermore, we reviewed the clinical features and the variants of all reported individuals with *PIGS* variants (Table 1). The phenotype of these individuals included hypotonia, severe global developmental delay, seizures, visual impairment, hand anomalies, and characteristic facial features. According to this report, the results of real-time PCR and western blotting showed a decrease in PIGS expression of up to 50% and a significant decrease in protein levels. In addition, flow cytometry analyses indicated that the individuals with *PIGS* pathogenic variants showed a reduced expression of GPI-APs on the cell surface (Nguyen et al., 2018).

**TABLE 1:** Summary of clinical features in patients with *PIGS* variants.

**References**	**Zhang et al.**			**Nguyen et al.**			
Patients	F1:?0.1	F2:?0.2	F2:?0.3	F3:?0.1	F3:?0.2	F4:?0.2	F4:?0.3
Types of variants	Heterozygous	Heterozygous	Heterozygous	Homozygous	Homozygous	Heterozygous	Heterozygous
	Variants	Variants	Variants	Variants	variants	variants	variants

*PIGS* variants	c.[148C > T]	c.[108G > A]	c.[108G > A]	c.[1316_1352delins]	c.[1316_1352delins],	c.[468 + 1G > C],	c.[468 + 1G > C],
	[1141_1164dup]	[101T > C]	[101T > C]	[1316_1352delins]	[1316_1352delins]	[923A > G]	[923A > G]

Gender	Male	Male	Male	Male	Male	Female	Female
GA weeks	40	35	35	41	38	19	13
Microcephaly	(−)	(−)	(−)	(+)	(+)	(−)	(−)
Hypotonia	(+)	(+)	(+)	(+)	(+)	NA	NA
Global developmental delay	(+)	(+)	(+)	(+)	(+)	NA	NA
CNS atrophy	Cerebellar	Cerebellar	Cerebellar	Diffuse cortical	Diffuse cortical	NA	NA
Hearing loss	(+)	(+)	(+)	(−)	NA	NA	NA
Visual impairment	(+)	(+)	(+)	(+)	(−)	NA	NA
Coarse facial features	(+)	(+)	(+)	(+)	(+)	(−)	(−)
Feeding problems	Oral feeding	Oral feeding	Oral feeding	Gastrostomy tube	Oral feeding	NA	NA
Recurrent respiratory infections	(+)	(−)	(−)	(+)	(+)	NA	NA
Seizures	Infantile spasms	Lennox–Gastaux syndrome	Febrile seizure	Intractable seizures	Intractable seizures	NA	NA
Seizure onset	2 months	8 months	1 year	NA	8 months	NA	NA
Treatment	(+)	(+)	(+)	(+)	(+)	NA	NA
Seizure-free	(−)	(−)	(−)	(+)	(−)	NA	NA
GPI-AP decrease	(+)	(+)	(+)	(+)	(+)	NA	(+)

*(+), features present; (−), features absent; NA, not applicable or not determined.*

The major symptoms of our patient were similar with the phenotypes described by Nguyen. No *PIGS* variant, except in our patient, has been reported with IS. IS is a specific type of seizure seen in an epilepsy syndrome of infancy. Approximately two-thirds of affected infants have a detectable underlying neurological abnormality, but the pathophysiological basis is not clear. Therefore, inherited GPI deficiencies should be considered in the differentiation of infantile spams from other disorders like West syndrome.

Furthermore, it is noteworthy that pyridoxine hydrochloride was reported to improve the seizures in patients with *PIGS* and *PIGO* variants (Kuki et al., 2013; Nguyen et al., 2018), and histone deacetylase inhibitor, butyrate, was able to control the intractable epilepsy of a child with *PIGM* variants (Almeida et al., 2007). Joshi et al. (2016) reported two brothers with *PIGA* variants and who had intractable epilepsy but became seizure-free on a ketogenic diet. Moreover, pyridoxine hydrochloride improved the seizures of one affected proband described by Nguyen et al. (2018). Unfortunately, there was no more follow-up therapeutic treatment in our case as the patient has died; more cases should be collected and researched to facilitate the establishment of personalized treatment methods for patients with GPI deficiencies.

## Conclusion

We identified novel *PIGS* pathogenic variants [c.148C > T (p.Gln50^∗^) and c.1141_1164dupGACATGGT GCGAGTGATGGAGGTG (p.Asp381_Val388dup)] in a Chinese boy with ISs, severe global developmental delay, hearing loss, visual impairment, hypotonia, and intellectual disability. The pathogenicity of this variant was proven by flow cytometry, in which the GPI-anchored proteins (CD59) and FLAER of this individual decreased. This case is the first report on *PIGS* pathogenic variants among the Chinese population. In patients with multiple exterior abnormalities combined with severe global developmental delay, intractable seizures, hearing loss, visual impairment, hypotonia, and feeding difficulty, GPI deficiency should be considered.

## Data Availability Statement

The datasets for this article are not publicly available due to concerns regarding participant/patient anonymity. Requests to access the datasets should be directed to the corresponding author.

## Ethics Statement

The studies involving human participants were reviewed and approved by the Ethics Committee of Xiangya Hospital, Central South University. Written informed consent to participate in this study was provided by the participants’ legal guardian/next of kin. Written informed consent was obtained from the minor(s)’ legal guardian/next of kin for the publication of any potentially identifiable images or data included in this article.

## Author Contributions

LZ contributed to clinical data collection and writing of the manuscript. XM and HL guided the completion of this manuscript. ZL and XJ participated in the flow cytometry analyses. BX and WX performed the DNA analysis. All the authors contributed to manuscript revision and read and approved the submitted version.

## Conflict of Interest

The authors declare that the research was conducted in the absence of any commercial or financial relationships that could be construed as a potential conflict of interest.
